# Updates on the risk factors of acute kidney injury after transcatheter aortic valve replacement

**DOI:** 10.15171/jrip.2017.03

**Published:** 2016-10-24

**Authors:** Wisit Cheungpasitporn, Charat Thongprayoon, Kianoush Kashani

**Affiliations:** ^1^Division of Nephrology and Hypertension, Department of Medicine, Mayo Clinic, Rochester, MN, USA; ^2^Department of Internal Medicine, Bassett Medical Center, Cooperstown, NY, USA; ^3^Division of Pulmonary and Critical Care Medicine, Department of Medicine, Mayo Clinic, Rochester, MN, USA

**Keywords:** Acute kidney injury, Transcatheter aortic valve replacement, Balloon valvuloplasty

**Implication for health policy/practice/research/medical education:**

In our recent updated studies, we found a significant association between periprocedural blood transfusion and acute kidney injury (AKI) following transcatheter aortic valve replacement (TAVR) with an overall 1.95-fold increased the risk of AKI. We also demonstrated that a transapical approach was significantly associated with increased AKI risk compared with a transfemoral approach. Nevertheless, the TAVR approach did not affect severe renal outcomes or long-term renal function. In addition, our meta-analysis demonstrated no significant association between contrast media volume and risk of AKI after TAVR. Thus, the dose of contrast media likely does not play a significant role in the pathogenesis of TAVR-related AKI. Growing knowledge of these risk factors of TAVR on kidney function will help improve preventive measures to improve patients’ outcomes.

## Dear Editor,

We agree with Onuigbo and Agbasi that previous studies have attempted to identify effective interventions to prevent postoperative acute kidney injury (AKI) events including cardiac surgery-associated AKI and most of them failed to be successful (1,2). In the setting of transcatheter aortic valve replacement (TAVR), we recently performed a comprehensive review of available data on incidence and risk factors of AKI after TAVR (3). We demonstrated that causes of AKI following TAVR are likely multifactorial including hypotension during rapid ventricular pacing for balloon valvuloplasty and valve deployment, use of contrast agents, and embolization resulting from the manipulation of catheters in the aorta of patients with diffuse atherosclerosis (4-6). However, we found that the data on effects of transapical (TA) vs. transfemoral (TF) approaches, contrast media and blood transfusion on AKI following TAVR were still controversial. Thus, we performed additional studies to evaluate these effects on AKI after TAVR, and ultimately to prevent AKI. We recently conducted a meta-analysis of 17 cohort studies with 5,085 patients assessing the risk of AKI in patients undergoing TA-TAVR vs. TF-TAVR (7). We found a significant association between TA-TAVR and a higher risk of AKI. However, the risk of severe AKI in patients who underwent TA-TAVR compared with TF-TAVR was not significantly higher. We subsequently performed a propensity-adjusted analysis of 366 patients undergoing TAVR to compare the incidence of post-procedural AKI following TA-TAVR vs. TF-TAVR (8). We confirmed that a TA approach was significantly associated with increased AKI risk compared with a TF approach. Nevertheless, the TAVR approach did not affect severe renal outcomes or long-term renal function. Regarding the effects of contrast media volume on TAVR-related AKI, we recently analyzed the data from 4 cohort studies comprised of 891 patients undergoing TAVR (9). Interestingly, our meta-analysis demonstrated no significant association between contrast media volume and risk of AKI after TAVR. Although the finding in this study might be limited by the lack of statistical power in detecting clinically meaning the effect of contrast volume on AKI, given a small number of included studies, these effects were small. Thus, the dose of contrast media likely does not play a significant role in the pathogenesis of TAVR-related AKI. Lastly, our recent meta-analysis of 16 cohort studies with 4690 patients assessing the risk of AKI after TAVR in patients who received a periprocedural blood transfusion (10). In this study, we found a significant association between periprocedural blood transfusion and AKI following TAVR with an overall 1.95-fold increased the risk of AKI. Growing knowledge of these risk factors of TAVR on kidney function will help improve preventive measures to improve patients’ outcomes.

## Authors’ contribution

All authors have contributed equally to the preparation of the manuscript.

## Conflicts of interest

The authors declare no conflict of interest.

## Ethical considerations

Ethical issues (including plagiarism, data fabrication, double publication) have been completely observed by the authors.

## Funding/Support

None.
